# Chitosan-Based Biocompatible Copolymers for Thermoresponsive Drug Delivery Systems: On the Development of a Standardization System

**DOI:** 10.3390/pharmaceutics13111876

**Published:** 2021-11-05

**Authors:** Lorenzo Marsili, Michele Dal Bo, Federico Berti, Giuseppe Toffoli

**Affiliations:** 1 Department of Chemical and Pharmaceutical Sciences, University of Trieste, Via Licio Giorgieri 1, 34127 Trieste, Italy; fberti@units.it; 2 Experimental and Clinical Pharmacology Unit, CRO National Cancer Institute IRCCS, Via Franco Gallini 2, 33081 Aviano, Italy; mdalbo@cro.it (M.D.B.); gtoffoli@cro.it (G.T.)

**Keywords:** thermoresponsive polymers, reproducibility, chitosan, drug delivery, smart drug delivery systems, poly-*N*-vinyl caprolactam

## Abstract

Chitosan is a natural polysaccharide that is considered to be biocompatible, biodegradable and non-toxic. The polymer has been used in drug delivery applications for its positive charge, which allows for adhesion with and recognition of biological tissues via non-covalent interactions. In recent times, chitosan has been used for the preparation of graft copolymers with thermoresponsive polymers such as poly-*N*-vinylcaprolactam (PNVCL) and poly-*N*-isopropylamide (PNIPAM), allowing the combination of the biodegradability of the natural polymer with the ability to respond to changes in temperature. Due to the growing interest in the utilization of thermoresponsive polymers in the biological context, it is necessary to increase the knowledge of the key principles of thermoresponsivity in order to obtain comparable results between different studies or applications. In the present review, we provide an overview of the basic principles of thermoresponsivity, as well as a description of the main polysaccharides and thermoresponsive materials, with a special focus on chitosan and poly-*N*-Vinyl caprolactam (PNVCL) and their biomedical applications.

## 1. Introduction

From a physiological point of view, only a few materials can be considered to be totally inert. Almost every known material contains different toxic components or irritating properties. A biomaterial is defined as a substance that can function as a part of a system that aims to treat or replace any tissue, organ or body function during a certain amount of time [1]. Moreover, during the setting of such materials, harmful effects can be caused by chemical reactions [2]. Williams provides a useful definition of biocompatibility, which is “*the ability of a biomaterial to perform its desired function with respect to a medical therapy, without eliciting any undesirable local or systemic effects in the recipient or beneficiary of that therapy, but generating the most appropriate beneficial cellular or tissue response to that specific situation and optimizing the clinically relevant performance of that therapy*” [3].

The biocompatibility of a material is assessed by establishing the relationship between the material and the organism, so that neither of them produces undesirable effects. In order to accommodate a substance in the class of biomaterials, it has to satisfy various prerequisites, including biocompatibility. The verification of this feature implies that its components must undergo different testing procedures, which are performed as recommended by different federations and organizations [4]. In general, for the appropriate evaluation of an experimental material, the safety for clinical application in humans is established throughout a sequence of research protocols, which have been described and regulated in many countries [1].

Considering that the term “biocompatibility” incorporates many different aspects, including physical, chemical and mechanical properties, as well as potential allergenic, mutagenic and cytotoxic effects, these evaluation protocols involve different areas, such as chemistry, biology and pharmaceutics, and include different studies ranging from virtual simulations to in vitro and in vivo assays and clinical trials [5]. Most medical studies focus on the toxicity of degradation by-products and the inflammatory reaction or immune response of the body when dealing with local implants or regional therapies. In the field of nanomedicine, the surface properties such as the roughness and surface energy are of major interest as they play a fundamental role in cell–material interactions, since they define the pattern of adsorption onto biological surfaces and the spatial orientation. Before approval, it is of major importance to monitor the interactions between the biomaterial and the proteins present in the physiological fluids that guide the inflammatory response, such as albumin, immunoglobin, fibrinogen and fibronectin [6]. Among biomaterials, a variety of polymers can be used for biological and medical applications, including in drug delivery scaffolds and the replacement of tissues, which are referred to as polymeric biomaterials [7,8,9,10,11,12]. These polymers can be of synthetic or natural origin, and they behave mechanically in a way that resembles natural tissues [9,10,11,13]. Natural polymers, such as polynucleotides, polypeptides and polysaccharides, are produced by living cells and are commonly used in medical applications as they allow for cellular adhesion and recognition, cell growth and differentiation [14]. Nonetheless, their biological application is hindered by the lack of control over their properties, which are not easily modified with processing. Synthetic polymers, on the contrary, can be produced industrially with control over several parameters, including morphology, mechanical properties and solubility. Notwithstanding the promising properties of polymers prepared for biological applications, their utilization is usually prevented by low rates of cellular adhesion and recognition and their lack of biodegradability [13].

In the first part of this review, we provide a description of the main polysaccharides and synthetic thermoresponsive polymers that have been proposed for drug delivery applications, with a specific focus on chitosan (CS). In the central part, we provide an overview on the basics of thermoresponsivity as well as a state-of the art description of synthetic thermoresponsive materials, with a detailed focus poly-*N*-Vinyl caprolactam (PNVCL) and its characterization. The final chapter is dedicated to the recent efforts on the production of chitosan-based thermoresponsive copolymers, as well as their most relevant application and physicochemical properties.

## 2. Polysaccharides: Natural Biocompatible Polymers

Polysaccharides, also known as polymeric carbohydrates, are natural polymers built from monosaccharides via O-glycosidic bonds that can be isolated from different sources, including animals, plants, algae and other microorganisms, such as bacteria and fungi [15]. Polysaccharides represent a broad class of materials with wide structural diversity and functional versatility, that derives from the abundant presence of functional groups along the molecular chains. The abundance of hydrophilic groups, mainly hydroxyls, carboxyls, carbonyls and amines, provides aqueous solubility and allow the possibility of adhesion and recognition between biological tissues via non-covalent interactions [16]. Due to their excellent adaptability to the cellular physiology, polysaccharides fall into the category “generally recognized as safe” (GRAS) defined under sections 201(s) and 409 by the Federal Food, Drug and Cosmetic Act, meaning that they are considered to be biocompatible, biodegradable and non-toxic materials [7,15,16]. 

Polysaccharides can be classified according to their chemical structure or composition, source, solubility and applications. Based on their chemical structure, polysaccharides are divided in homoglycans, consisting of a polymer made by the repetition of a single type of monosaccharide, and heteroglycans. Another useful way to classify polymers is by considering their positive charge, as it defines the interactions between polymer and biological tissues. Following recent advances in technology and processes, polysaccharides have been established as important alternative materials in various applications for their versatility, low cost and ease of use [17]. On account of these advantages, many polysaccharides such as CS, hyaluronan, dextran, cellulose, starch, pectins and pullulan have been extensively used as drug-delivery materials [18]. Their inherent bioactivities allow one to modulate the response of the immune system and to include receptor recognition and binding, site-specific enzymatic degradation, mucoadhesion and transport and environmental triggering [15]. A list of biomedical applications for polysaccharides is reported in Table 1 [18,19]. In addition, upon contact with water, hydrophilic polymers can form hydrogels, solid-like materials composed of water and a three-dimensional polymer network. Due to their high water content, which can be up to 99% of their total mass, hydrogels provide tissue-like properties that have been studied extensively as drug delivery systems for their biocompatibility and ability to encapsulate drugs [20].

Through chemical modifications, polysaccharides that undergo gelification can be modified in order to provide the desired properties as a building block for the development of drug delivery systems. Recent advances have led to the development of smart platforms, provided by both bulk and nano-sized hydrogels (micro- and nanogels), which are able to respond to a specific internal or external trigger due to their chemical structure. Due to their ability to respond to different and multiple stimuli, these systems cover a wide range of applications. These promising structures are usually referred to as “smart drug delivery systems” and their application is still confined in pre-clinical trials due to a lack of toxicity assessments and standardized manufacturing methods [21].

In drug delivery systems based on polysaccharide particles, the polymeric biomaterial provides the scaffold in which the drug is absorbed or bound to the internal or external surface. This increases drug stability in unsuitable environments and can also enhance the solubility, thereby providing enhanced permeation and retention at the site of interest [15]. Furthermore, the functional moieties on the carbohydrate backbone can be modified chemically in order to conjugate imaging probes and targeting agents, such as antibodies, in order to provide prolonged circulation times and site-specific accumulation activities [16].

One of the main drawbacks to the utilization of polysaccharides in drug delivery comes from the variability of their properties. Naturally sourced polymers usually have broad molecular weight distributions that can significantly vary between each batch [7], and their properties cannot be changed easily due to the lack of solubility in most organic solvents [22]. The molecular weight, along with the molecular composition, is one of the main determinant factors of the polysaccharide behavior in physiological environments, which determines the chain flexibility, intra- and intermolecular interactions, scaffold shape, surface charge, drug loading capacity and polymer solubility in blood and plasma. These physicochemical properties establish the types of interactions with the proteins and their immunogenicity. Consequently, the controls regarding the development of polysaccharide drugs or drug scaffolds start from the standardization of the synthesis process and product characterization, since the preliminary stages of development occur in the laboratory. The need for improved quantification and standardization of their physicochemical properties has posed considerable problems for the development of polysaccharide-based drug delivery systems with well-defined characteristics that can reach clinical approval. As a general rule, their translation into clinical studies requires more insight into their drug release properties, targeting and therapeutic efficacy, and a more detailed description of their degradation profile. Despite this, some formulations have entered clinical trials with different types of delivery routes, including percutaneous or intracoronary injection, intravenous infusion, inhalation, xenotransplantation, intravesical instillation and topical skin application [16]. The first polysaccharide that entered a phase I clinical trial was dextran in 1993, under the name AD-70. This polysaccharide was developed in Germany in 1993 by Danhauser-Riedl et al. for the treatment of refractory solid tumors [23]. Over the years, CS (Millican, 2006, small HCC) [24], hyaluronic acid (Radiaplex, 2007, radiation dermatitis) [25], cyclodextrins (CALAA-01, 2008, solid tumors) [26] and alginate (DIABECELL, 2009, type I diabetes) [27] have also reached the clinical trial stage, although the development stage of treatment has never advanced beyond phase III.

## 3. Chitosan

**Figure 1:**
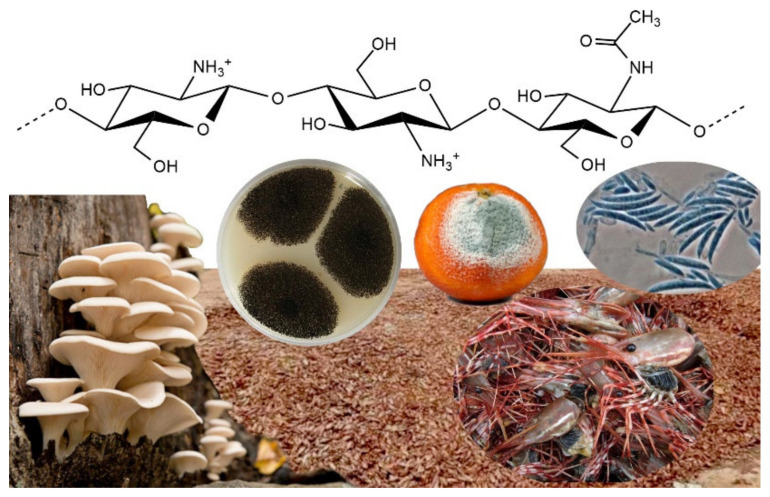
Natural sources of chitosan: animals (seafood waste, mainly crabs and shrimps) and mushrooms (from left to right: Pleurotus Ostreatus, Aspergillus Niger, Penicillum Citrinum, Fusarium Oxysporum).

CS is a biodegradable and biocompatible polysaccharide consisting of a linear polymer of β-(1→4)-linked glucosamine (2-amino-2-deoxy-glucopyranose). The polymer is obtained through N-deacetylation of chitin, a natural polymer contained in the cell walls of fungi and arthropods, such as crabs and shrimps (Figure 1). Initially, CS was thought to be an easily accessible substance obtained from seafood industry waste. Another natural source for CS is mushrooms, which is considered to be safer for biomedical applications as it usually exhibits a narrower molecular weight distribution compared to CS obtained from animal sources [28]. To date, CS is one of the most cited polymers across a very extended scientific range that includes food science and environmental, biopharmaceutical and biomedical applications [29]. This polymer first appeared in a study by Clark and Smith that described the X-ray investigation of N-deacetylated chitin fibers extracted from lobster tendons [30]. By early 2020, the polymer had been mentioned in about 75,000 papers (source: Scopus, November 2020).

**Figure 2:**
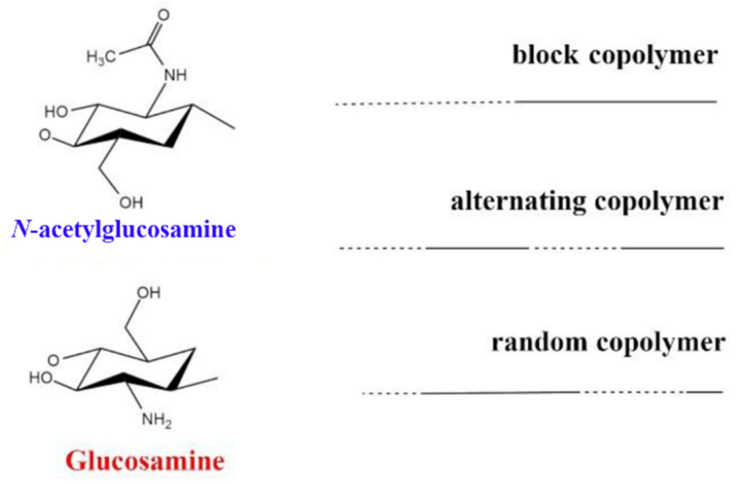
Schematic representations of three possible conformations for chitosan copolymers. The chemical structures of the two repetitive units are represented on the left.

Among all natural polymers, it is uniqueness due to the presence of the glucosamine monomer, which can be either partially or fully deacetylated. This structural characteristic is the basis of its remarkable biophysical properties. In aqueous solution, the polymer behaves similarly to a linear polysaccharide with positive charge density at low pH, making it the only positively charged high molecular weight polysaccharide produced on an industrial scale [29]. Commercial CSs are produced in high yields with average molecular weights ranging between 3.8 and 2000 kDA and with degrees of deacetylation (DDA) ranging between 66 and 95% [29,31]. For this reason, it is more appropriate to talk about “chitosans” rather than chitosan, as the term implies a series of copolymers that differ in their percentages of fractions (the deacetylated degree, DDA), but also in the distributions and lengths of their comonomer sequences (Figure 2). This copolymer characteristic has been underestimated in the literature, where it has been compared to other natural polymers, such as alginates. The two monomers, glucosamine and N-acetylglucosamine, display different solution properties, as the first provides the ionic characteristic and the second is slightly hydrophobic. This difference is amplified by the presence of a block-type sequence and can have dramatic consequences on chain conformation and aggregation [29]. Due to the presence of amino groups, CS has intrinsic acidic properties and its pKa range is between 6.17 and 6.51, depending mainly on the molecular weight and DDA [32]. Consequently, the polymer is not soluble at neutral pH and its solubility is troublesome when the pH is kept over 6, especially at high molar masses [29]. Alkyl and acyl side chain derivatives of CS have been prepared for the fabrication of copolymers with increased hemocompatibility and water solubility [33,34]. This kind of modification can be achieved via atom transfer radical polymerization (ATRP), chemical treatment, biocatalysis and ring opening [33]. Many studies have reported the utilization of derivatives, such as pegylated CS [35,36], trimethylammonium [29,37,38] and polyvinylic [39,40] and polyacrylic [41] derivatives for the utilization of CS at physiological pH. Other studies have reported chemical and physical treatments in order to achieve low molecular weight (LMW) CS for enhanced solubility and faster degradation [42,43]. CS has been extensively studied as a polycationic biomaterial for its ability to adhere to negatively charged surfaces. It was originally conceived as an excipient in solid dosage form and used as a coating, tablet binder, lubricant, viscosity-increasing agent, disintegrant and mucoadhesive. In relation to synthetic cationic polymers, CS has very low immunogenicity and can be used as a bioadhesive for negatively charged mucosal cells and to increase transport and retention properties [44].

The first study reporting the utilization of CS for drug delivery was published by Henriksen in 1993 [45]. The mucoadhesive properties of CS have been demonstrated by its ability to adhere to porcine gastric mucosa in vitro [46]. It is believed that the positive charge enhances the opening of the epithelial junctions and widens the paracellular pathways [38]. Based on these considerations, CS has been studied as a scaffold polymer for both hydrophilic and hydrophobic drugs [47,48]. Although the initial attention was focused on the formulation of microparticles [29,38], the attention has rapidly shifted towards the fabrication of nanoparticles, along with the increase in nanomedicine. The choice between a nano- or micrometric-based formulation is defined by the type of application. In the case of pulmonary delivery, microparticles have better efficacy [29], while nanoparticles are usually preferred for intravenous administration, unless the formation of an embolus is intentional, such as in chemoembolic treatment [49]. Furthermore, it has been suggested that CS is a valuable polymer for gastrointestinal and small intestine drug delivery [37]. To date, thanks to the opportunities provided by the developing of nanostructure systems and by modifying the polymer backbone with ionic or hydrophobic moieties, there are thousands of different formulations for dental, buccal, nasal, gastrointestinal, colon, mucosal and gene delivery [50].

Oral delivery is the most common administration method of CS formulations. However, this application is limited for several types of drugs that can cause irritation to the gastric mucosa and by the slow onset of action of the adminisetred drugs [51]. In recent years, the utilization of hydrogels [52,53], nanoparticles [54,55] and micelles [56,57,58] has allowed the develop oral formulations that are able to increase the mean residence time in the absorption microenvironement due to the pH-responsive and mucoadhesive properties of CS. Furthermore, CS derivatives have been applied for the treatment of diabetes [52,53], cardiac diseases and cancer therapy [24,59]. CS–dextrane sulfate hydrogels have been applied for pH-controlled release of insulin at acidic and neutral pH [52,53]. The utilization of PMMA-CS-PEG micelles has been reported for the pH-controlled delivery of metropolol tartrate [60]. The utilization of CS–graft–stearic acid micelles for oral delivery of doxorubicin (DOX) has been studied in vivo. Polymeric micelles formulations prolong the half-life ciculation and DOX bioavailability [59].

Buccal drug delivery of CS and its derivatives has been studied in order to exploit the polymer’s mucoadhesive properties. Buccal delivery avoids gastric degradation [61] and has been applied in the form of patches, hydrogels, buccal tablets and wafers for the treatment of cardiac diseases [62] and for the administration of anestetichs [63] and proteins [64].

Many studies have reported on the fabrication of CS-based NPs for nasal route delivery. It is has been reported that nasal delivery increases the bioavailability of the drug, especially for local administration, and represents an efficient strategy for brain targeting [33]. Casettari et al. reported that mPEG-CS composites efficiently promote absorption in nasal drug delivery and can extend the solubility of CS in water up to basic pH (9.6) [35,65]. The intramuscolar delivery of chitosan-derived NPs and chitosan–pullulan composites loaded with diphteria toxoid was compared to nasal delivery by Cevher et al. [66]. N-trimethylaminomethylmethacrylate chitosan–ovoalbumin particles have also been applied for the nasal delivery of vaccines [67]. The particles exhibited quick distribution and elicited an immune response through the nasal route. These studies showed that the nasal route may represent a valuable alternative to intramuscolar injection in vaccine delivery.

In gene delivery, CS is regarded for its ability to form a complex with DNA through electrostatic interactions in acidic solutions [68]. The formation of CS and DNA nanoparticles is achieved by coacervation via eletrostatic interactions between the amino groups of the polymer and DNA phosphate groups. The polymer was first described as a delivery vector for plasmids [69]. The efficiency of transfection depends on the molecular mass, pH and DDA [50]. Nonetheless, the major limitations include the low solubility of CS at physiological pH, premature release inside the cytoplasm and low stability of CS–DNA complexes after cellular uptake. In recent times, chemical modifications, such as PEGylation, have been used to improve the solubility of CS. However, reductions in CS’s positive charge via chemical modification affect the interactions between CS and various genes. Furthermore, siRNA modifications can increase the stability of CS-siRNA nanoparticles but compromise the siRNA efficacy [70].

Colon-specific delivery of CS is widely regarded due to the high solubility of the polymer in gastric fluids. As with most polysaccharides, the polymer exhibits degradation in the colon [50]. In vitro studies have demonstrated that CS spheres are able to retain the drug at acidic pH and release it in the presence of the colonic microenvironment [71]. The polymer has been studied for the delivery of insulin [72], antibiotics [73,74] and anti-inflammatory drugs [75,76,77] and in the treatment of colon cancer [71,77].

CS has been also extensively used as a medicament in various fields of medicine and dentistry as a wound-healing material [78]. CS has been used in endodontics as an anti-inflammatory for periapical lesions [50,79]. It has been reported that the utilization of CS linked to synthetic dental materials may be advantageous due to its bacteriostatic or mycostatic activity. Due to its high water uptake capacity, CS is also considered a material of interest for wound dressing application. The possibility of combining CS’s properties with other polymers via ATRP and click chemistry has allowed further increases in water uptake capacity. Tirino et al. reported on the synthesis of CS-crosslinked PEO via click chemistry using copper-catalyzed cycloaddition. The utilization copolymer was able to increase the water uptake rate by 940% [80].

CS-based formulations have been documented to increase drug solubility and biovailability and to reduce toxic effects. The polymer has entered clinical trials for the treatment of small HCC (Millican, 2006) [24]. The conjugation of chemotherapy drugs usually occurs in the amino acid by means of weak interactions or covalent bonds, depending on the required flexibility of the system and the application. A common approach involves covalent conjugation via the utilization of a succinic anhydride spacer [47]. Another approach for the encapsulation of chemotherapeutics involves the formation of micelles, or core–shell nanoparticles, which has been applied for both hydrophilic drugs (e.g., DOX) and hydrophobic drugs with low water solubility, such as 5-fluorouracil [81], paclitaxel [47] or methotrexate [82]. The polymer has entered clinical trials for the treatment of small HCC (Millican, 2006) [24]. Among the various remakable properties of chitosan, the polymer is also known for its bioactivity as an antioxidant, antimicrobial and antifungal compound [38]. The killing potential is based on the electrostatic interaction with the negatively charged microbial surface, which dramatically affects the bacterial vitality [29].

## 4. Synthetic Polymers as Biomaterials

Synthetic polymers represent the broadest and most diverse class of biomaterials and are available in a variety of compositions for any kind of application [83]. Compared to natural polymers, synthetic biomaterials have more batch-to-batch uniformity and more predictable properties. Their main advantage is that they provide tailored property profiles for specific applications, including the ability to respond to an external stimulus, such as a change in concentration, pH or temperature. Furthermore, their final properties can be easily tuned depending on the desired application by controlling their chemical structure and conformation through the manufacturing process. In order to provide efficacy, synthetic polymers require specific biological, chemical, physical, biomechanical and degradation properties.

The first synthetic polymer used in biological applications was poly(-glycolic acid) (PGA) in 1967 [84]. Since then, technological progress has allowed significant steps to be made in the development of biodegradable polymeric materials for biomedical applications. More recently, natural and synthetic materials have been combined into hybrid materials in order to enhance their biological activity [85]. This has allowed the development of degradable polymeric biomaterials as suitable candidates for drug and gene delivery vehicles, scaffolds for tissue engineering and transient implants for orthopedic and related medical applications.

## 5. Thermoresponsivity

**Figure 3:**
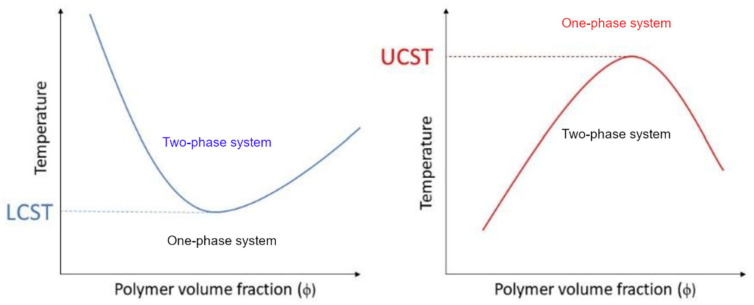
Temperature vs. polymer volume fraction (ϕ). Schematic illustration of phase diagrams for the polymer solution’s lower critical solution temperature (LCST) (**left**) and upper critical solution temperature (UCST) (**right**).

Stimuli-responsive materials can alter their physicochemical properties upon exposure to external stimuli. Among these, thermoresponsive or temperature-responsive polymers are characterized by a drastic and discontinuous change in their physical properties with temperature. Accordingly, thermoresponsive polymers display a miscibility gap in their temperature–composition diagram at a critical solution temperature (Figure 3).

**Figure 4:**
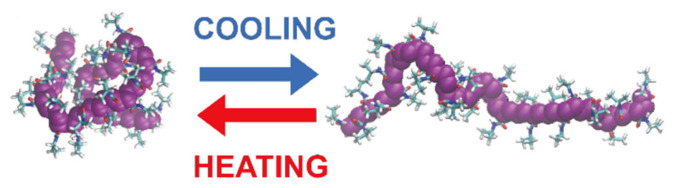
Schematic representation of a coil-to-globule lower critical solution temperature (LCST) transition. Adapted with permission from [97], copyright by Elsevier, 2019.

If solubility is reached upon heating, this point is called the upper critical solution temperature (UCST). If the polymer becomes insoluble over a critical temperature, the point is called the lower critical solution temperature (LCST) [86,87,88,89,90]. However, their behavior can be quite complex to describe, as a polymer can exhibit both behaviors depending on many different factors, including the molecular mass, polymer concentration, termination groups and the presence of cosolutes. Most of the thermoresponsive polymers are amphiphilic, possess both hydrophobic and hydrophilic segments and are able to self-organize in solution. As a result, the solvent–polymer mixture can result in a self-organized micelle, a gel, a globule, a coil or a two-phase system, depending on the conditions [91]. In water, these conformational changes arise from the balance between the intra- and intermolecular hydrophobic attraction of the polymeric backbones and the hydrophilic interactions between the hydrophilic groups and the solvent [92]. When a thermoresponsive polymer is completely dissolved, the hydrophilic groups (carboxylic acid, hydroxyl and amines) interact with the solvent molecules via hydrogen bonding. As a result, the polymer is solvated and the system appears as a clear solution. However, hydrogen bonding is only effective at low temperatures. When temperature is increased, the water is partially displaced from the polymer coil and the interaction is weakened. On the contrary, hydrophobic interactions follow the opposite trend as they tend to increase at higher temperatures [93]. If the interactions between the polymers become more favorable, the polymer undergoes a LCST transition and its conformation changes from coil to globule (Figure 4) [91,94]. At this point, particle aggregation usually results in visible turbidity. For this reason, the critical LCST point is frequently referred to as the “cloud point” [95], although this definition is imprecise [96]. A more detailed description of the difference between the LCST and cloud point will be provided in Section 7.2.

When the polymeric chains cause the complete displacement of the solvent, phase separation occurs. The free enthalpy of mixing ($\Delta G_{mix}=\Delta H_{mix}-T\Delta S_{mix}$), which accounts for enthalpic (Δ*H_mix_*) and entropic (Δ*S_mix_*) contributions, establishes whether UCST or LCST miscibility gaps occur. LCST transitions are associated with an unfavorable entropic effect. A decrease in solubility with a rise in temperature is originated with a negative, exothermic enthalpy of mixing. At a critical point (T_c_), phase separation occurs if the favorable energy effect overcome is overcome by a negative entropy term [98]. On the contrary, it is agreed that a UCST arises from strong polymer–polymer and solvent–solvent interactions compared to weak polymer–solvent interactions. Accordingly, the hydrophobic effect is more dominant in the LCST transition. According to Seuring et al., UCST behavior in water is quite common but was rarely observed under physiological conditions [99]. Since the hydrophobic effect is considered to be entropy-driven, UCST behavior is usually considered to be an enthalpy-driven transition [100]. However, a recent study described the hydrophobic effect as a far more complex process [101]. The hydration shell of small hydrophobic solutes is commonly described as a clathrate, ice-like structure, characterized by strong hydrogen bonding interactions between water molecules, which account for favorable enthalpic contributions and a distinct loss in entropy. However, the hydration of extended non-polar planar surfaces involves the formation of different structures of the hydration shells, in which unsatisfied hydrogen bonds are directed towards the hydrophobic surface [102]. The overall positive entropic contribution is generally assigned to the demixing between the two phase systems that breaks the organized water structures around the hydrophobic surfaces. Computational simulations have demonstrated that some hydrophobic interactions between ligands and receptors are enthalpy-driven, as the main contributions come from the expulsion of the disorganized water from the receptor cavity [103].

A starting approach to describe the behavior of polymer solution is the method developed by Flory and Huggins in 1942, known as the Flory–Huggins theory [104,105]. This theory employs a lattice model of the thermodynamics of polymer solutions, which takes into account the great dissimilarity in molecular sizes. The thermodynamic quantities of the solution are derived from a reduced Gibbs energy parameter, *χ*, and a simple concept of combinational entropy of mixing [106]. The *χ* parameter is calculated from Equation (1):

(1) $$\chi =\frac{z}{k_{B}T}(w_{12}-\frac{w_{11}+w_{22}}{2})$$

in which the left term is the energy increment for every monomer–solvent contact and involves three enthalpic contributions: a solvent–solvent interaction (*w*_11_), a monomer–monomer interaction between different chains (*w*_22_) and a monomer–solvent (*w*_12_) interaction. In the right term, *z* is the coordination number, *k_B_* is the Boltzmann constant and *T* is the temperature of the system. Consequently, the *χ* parameter is a dimensionless quantity that takes into account the reduced solubility of polymers at low temperature. The FH theory led to Equation (2) for the free energy of mixing:

(2) $$\Delta G_{mix}=RT\left[n_{1}ln\phi_{1}+n_{2}ln\phi_{2}+n_{1}\phi_{2}\chi \right]$$

The FH theory also led to the following equation (Equation (3)) for the Δ*G_mix_* normalized per lattice site:

(3) $$\frac{\Delta G_{mix}}{Nk_{B}T}=\chi \phi_{1}\phi_{2}+\frac{\phi_{1}}{x_{1}}ln\phi_{1}+\frac{\phi_{2}}{x_{2}}ln\phi_{2}$$

in which *ϕ* and *x* are, respectively, the volume and the molar fractions of the two-component system. From a qualitative point of view, the Flory–Huggins expression provided an adequate description of the main phenomena associated with the thermodynamic behavior of polymeric solutions [98]. The *χ* parameter considers the dramatic change of solubility with the lowering of temperatures, which can result in phase separation. Moreover, it is suitable for the description of the phase separation observed at UCST-critical points. Since the FH does not involve steric interactions, the temperature at which polymer–solvent interactions are balanced by long-range forces between polymer molecular segments is called the Flory or theta temperature *θ*. Under such conditions, the polymer is at the edge of solubility and exists in the form of a statistical coil, the solution is considered to be ideal and there is no enthalpy of mixing. Consequently, there is no excluded volume effect due to polymer expansion. The solvent at the theta temperature is also called the theta solvent [107]. At temperature *θ*, the second virial coefficient (*B*) of the osmotic pressure is zero (Equation (4)):

(4) $$\frac{\Pi }{RT}=\frac{c}{M}+Bc^{2}+B_{3}c^{3}+\cdots$$

Virial coefficients are functions that describe the deviations from the ideal gas law. If we consider that the chemical potential is related to the osmotic pressure by the simple relation reported in Equation (5):

(5) $$\Delta \mu =-v_{s}\frac{\Pi }{RT}$$

we can conclude that *B* is connected to the excess chemical potential and depends on the square polymer concentration. Its value gives a representation of the deviation of the solution behaviour from the ideal scenario due to polymer–solvent interactions (Equation (6)):

(6) $$B=-\frac{\Delta \mu_{exc}}{v_{1}c^{2}}$$

Since *B* reflects the binary interactions between solvent molecules and segments of the polymeric chains, it is connected to the behavior of the polymer in solution. When *B* > 0, polymer–solvent interactions are favorable and the solvent is referred to as “good”. In this condition, repulsive forces between the polymer segment promote the swelling of the chains into the solution. When *B* < 0, solvent–polymer interactions are unfavorable and the solvent is referred to as “poor”. Therefore, the polymeric chains shrink as they attract each other. The Huggins constant *χ* also depends on the quality of the solvent according to the following relation (Equation (7)):

(7) $$lna_{1}=\frac{\Delta \mu_{1}}{RT}=\ln\left(1-\phi_{2}\right)+\left(1-\frac{1}{N}\right)\phi_{2}+\chi \phi_{2}^{2}$$

in which *N* is the degree of polymerization of the polymer. Here, *θ* is evaluated by plotting the value of second virial coefficient in order to find the zero value, according to Equation (8):

(8) B=12−χv1ρ22

in which *ρ*_2_ represents the density of the polymer. According to these equations, good solvents are obtained if *χ* > 1/2 and poor solvents are obtained if *χ* < 1/2. The FH parameter for polymer–solvent pairs is extracted at infinite dilution [108]. For his fundamental achievements, Flory was awarded the Nobel Prize in Chemistry in 1974.

However, despite the remarkable achievements of the FH theory, the assumption of ideal polymeric chains has been demonstrated to be the reason for its limited application. The FH approach does not account for the change in conformation nor for the volume changes of the polymer associated with different phenomena, including LCST transitions. From a quantitative point of view, the main limit of the theory was the overestimation of the value for *χ*, which was in contrast with the definition in terms of the contact energy. Moreover, the theory was based on the regular solution assumption, for which the entropy of mixing is equal to that of an ideal solution of the same composition, but it is not ideal due to the non-zero enthalpy of mixing. This assumption was not compatible with the dependency of *χ* with temperature. During the 1960s, the free energy parameter was redefined with a contribution of a non-combinatorial entropy of mixing: $\chi =\chi_{H}+\chi_{S}$. A further advance in the theory was provided by Patterson et al. [109], who provided a simple explanation for LCST by considering the free volume in the FH theory. The free volume (*v_f_*) is the measure of the quantity of space available in which polymer chains can change their conformation. The free volume can be formulated as $v_{f}=v-v_{o}$, in which *v* is the volume occupied by the polymer at a certain temperature and *v_o_* is the limiting occupied volume or the incompressible molecular volume, which depends on the temperature, pressure and concentration. The new theory provided a suitable explanation for the calculation of the parameter *χ* under different conditions, including its dependence on the concentration and the related mixing temperatures Δ*H_mix_* [98]. The new formulations included a new expression of the *χ* parameter as reported in Equation (9), which included a solubility parameter:

(9) $$\delta_{2}={\left(\frac{\Delta E_{2}^{vap}}{v_{2}}\right)}^{\frac{1}{2}}$$

This included the energy of evaporation $\Delta E_{2}^{vap}$ and the molar volume of the polymer. The free energy parameter became (Equation (10)):

(10) $$\chi =v_{2}\frac{{\left(\delta_{1}-\delta_{2}\right)}^{2}}{RT}=\frac{z\Delta w}{k_{B}T}$$

According to Equation (10), the change of *χ* with rising temperature is caused by the rapid decrease in *δ*_1_ in comparison to the slow decrease in the polymeric *δ*_2_. At higher temperatures, *χ* follows an opposite trend, as indicated by the FH theory, and increases with increasing temperature. The critical point at which this change of trend is observed is *T_c_*, corresponding to the LCST of the system. The inherent Δ*H_mix_*, derived from the Gibbs–Helmotz equation, with the assumption that all temperature variations are caused by *δ*_1_, is the following, as reported in Equation (11):

(11) $$\Delta H_{mix}≅2V_{mix}\delta_{1}\delta_{2}\left(\delta_{1}-\delta_{2}\right)T\frac{\partial \delta_{1}}{\partial T}$$

which predicts that Δ*H_mix_* can be positive or negative according to the difference between *δ*_1_ and *δ*_2_. According to this model, negative enthalpies of mixing are expected even for chemically similar systems. This should be considered to be the opposite of the “like dissolves like rule”, as a chemically similar solvent is not expected to be the best solvent for the dissolution of a polymer [109]. Another formulation, which includes the results of the Patterson and Flory, isreported in Equation (12):

(12) $$\chi =\nu_{12}\frac{\Delta E_{1}^{vap}}{RT}+\frac{C_{p1}}{2R}\tau_{12}$$

Here, *C_p_*_1_ represents the configurational heat capacity of the solvent, *ν*_12_ is a term related to the molecular differences in cohesive energy and size between polymer and solvent and *τ*_12_ is the expansivity or the difference in free volume between polymer and solvent. While the first term decreases with increasing temperature, the second temperature follows the opposite trend. The free volume term contributes to Δ*G_mix_* and always leads to a LCST transition, even when *ν* is zero. According to the free volume theory, since *χ* is increased by both lowering or increasing the temperature, phase separation can be reached by exceeding a *χ* critical value. Accordingly, two-phase polymer–solvent systems can display both LCST and UCST. The free volume theory of liquids predicts that all systems exhibit LCST, while UCST is caused by unfavorable positive mixing temperatures. For a polymer–solvent system displaying both LCST and UCST, the minimum value of *χ* provides the best solvent quality [98].

A more recent phenomenological analysis of the miscibility behavior, although general, differentiates between three types of limiting critical miscibility behavior. This approach is based on the definition of an interaction parameter *g*(*T*,*ϕ*), which can be modified to account for both the concentration and temperature dependence of the miscibility gaps. The g parameter is derived from the Flory–Huggins–Staverman (FHS) expression for the Gibbs free energy of mixing, as reported in Equation (13):

(13) $$\frac{\Delta G_{mix}}{NRT}=\frac{\phi_{1}}{m_{1}}ln\phi_{1}+\sum \frac{\phi_{2i}}{m_{2i}}\phi_{2i}+\phi_{1}\phi_{2}g\left(T,\phi_{2}\right)$$

In this equation, the first two terms represent the ideal part of the entropy of mixing. This combinatorial part is based on a similar lattice model in which m represents the number of lattice sites occupied by a molecule. The FHS model shows that the combinatorial entropy of mixing is smaller for polymers in relation to small molecules. In this condition, miscibility gaps can form easily due to small interactions. For this reason, it is standard practice to use the interaction parameter *g* for phase diagram modeling. In Equation (14), *ϕ*_1_ and *ϕ*_2*i*_ represent the volume fractions of the solvent and the number of species present in the polymer, respectively, while *ϕ*_2_ = Σ2*i* [110,111,112]. The interaction *g* is generally represented by a second-order polynomial in *ϕ*_2_, as reported in Equation (14):

(14) $$g\left(T,\phi_{2}\right)=g_{0}+g_{1}\phi_{2}+g_{2}\phi_{2}^{2}$$

The dependency from the temperature [112] can be describedin a similar way by Equation (15):

(15) $$g\left(T,\phi_{2}\right)=g_{0}+\frac{g_{1}}{T}+g_{2}T$$

**Figure 5:**
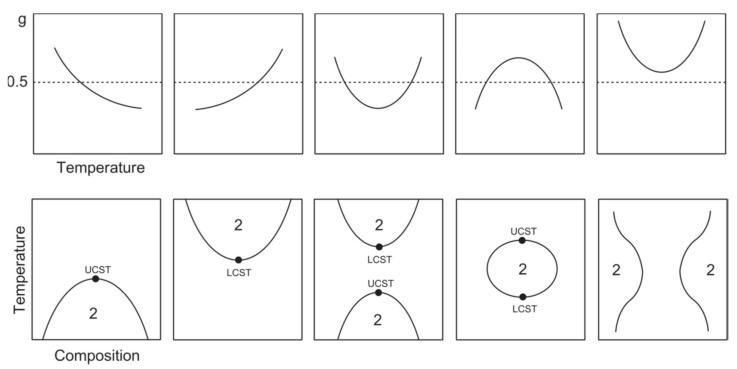
Correlation between the temperature dependence of the interaction parameter g and the type of miscibility gap. The number 2 indicates the presence of a two-phase region. Reprinted with permission from [99], copyright 2012, John Wiley and Sons.

In some cases, a polymer can show both UCST and LCST due to the complex temperature dependence of the interaction parameter, as shown in Figure 5. The values of *g*_1_ and *g*_2_ at the consolute point determine the type of phase behavior [110]. The classical Flory–Huggins miscibility behavior (type I) is observed if the following conditions are satisfied (Equation (16)):

(16) $$g_{0}\leq \frac{1}{2}+g_{1}\;\mathrm{and}\;g_{2}\geq g_{1}-\frac{1}{6}$$

A complete description of the theory is reported by Solc et al. [113]. In general, it is a strong assumption to assume a polymer solution as strictly binary. Even with a small and narrow distribution, it is impossible to produce polymers with a uniform molar mass. A polymer solution should be considered to be a quasi-binary mixture of polymers with different molecular masses. The FHS has been previously extended to account for polydispersity [114]. Under these conditions, the miscibility gap is shifted towards different temperature values and polymer concentrations. In addition, three-phase equilibria can appear and the curvature of the LCST curve can change. However, for the sake of simplicity, polymer–solvent mixtures will be referred to as binary mixtures in this review. In organic solvents, the g parameter is calculated on the assumption that most of the molecules interact via simple van der Waals forces. In water, these assumptions are not valid, since the polymer needs to contain polar groups that interact via polar interactions and hydrogen bonding.

These interactions exhibit a stronger and non-monotonic temperature and concentration dependence. In some cases, the strong concentration dependence can lead to an off-zero critical point for infinite molar masses. Since this discovery, polymers have been divided into three different types according to their phase behavior (Table 2). For type I polymers, also known as the “classical” type, the position of T_c_ is shifted with the increase in the polymer chain length—and consequently with the increase in the average molecular mass—towards a lower polymer concentration. At the limit of the infinite chain length (N→∞, or m→∞), the critical polymeric concentration at the ϑ temperature is zero. On the other hand, type II is characterized by a single off-zero critical polymeric concentration, ϕ_C_=0, which occurs under non-ϑ conditions. For a polymer characterized by type II behavior, the LCST critical point is almost independent of the polymer chain length. Finally, type III exhibits one zero-limiting critical concentration and two off-zero-limiting critical concentrations. The first critical point is usually exhibited at low polymer concentrations and the polymer behaves as described by the Flory–Huggins. The critical point shifts to zero concentration and to the ϑ temperature for N→∞. The other points are not influenced by the length of the polymeric chains at high polymer concentrations [110,111,112,113]. Examples of these include the LCST values of PNVCL (type I), poly-N-isopropilacrylamide (PNIPAM, type II) and poly(vinylmethyl ether) (PVME, type III).

## 6. Thermoresponsive Polymers for Biomedical Applications

The analysis of polymer behavior in water is fundamental in order to assess the potential for biological applications. Since cloud points arise from the balance between hydrophobic and hydrophilic interactions, every polymer with a zwitterionic character can theoretically exhibit a cloud point in water if appropriate conditions are reached. In water, LCST-type polymers are easily solvated via hydrogen bonding and polar interactions. Conversely, UCST behavior is less common in water [99,115] and it has been observed more frequently in organic or organic–water solutions [116,117,118,119,120,121,122,123]. Since UCST polymers rely mainly on non-polar interactions, their behavior can be strongly influenced by the presence of salts. UCST in organic solvent has been observed for common industrial polymers, such as polypropylene in n-hexane, polystyrene in butylacetate or polyethylene in diphenylether [124]. In some cases, both LCST and UCST have been observed in polymers of industrial relevance, such as polyethylene oxide (PEO), polyhydroxymethylmethacrylate (PHEMA) and polyvinylmethylether (PVME). However, the UCST of those polymers is usually observed outside the range of 0–100 °C. In other cases, cloud points have been determined under extreme experimental conditions or by exploiting salting-out effects using high salt concentrations (e.g., polyacrylic acid (PAA) in water) [125].

Thermoresponsive polymers are considered to be a “smart” class of materials that can be used in a wide range of applications. Their biological advantage consists of the presence of a desirable sharp transition. However, in order to be used in the biological field, thermoresponsive polymers must possess a cloud point at a temperature close to the normal human physiological temperature, which is usually between 36.5 and 37.5 °C, although this can oscillate over a wider range between 33.2 and 38.2 °C [126]. Therefore, synthetic procedures require fine optimization in order to ensure control of all structural parameters that can influence the positions of LCST and UCST points, such as the chemical structure, molecular weight and polymer morphology (linear, branched, star-shaped, comb, brush, network, dendrimer). This also applies to the preparation of copolymers. The possibility of combining different polymers together has provided thermoresponsive properties to other polymers of biological interest. This has increased the biodegradability of synthetic compounds by conjugating them with natural biopolymers and has allowed the combination of polymers or nanoparticles with different type of responsivity (pH, magnetic, concentration) for the preparation of dual- and multi-responsive smart delivery devices.

To date, hundreds of thermoresponsive polymers have been developed for various applications in the biological field, including tissue engineering, bioseparation and drug and gene delivery. Among these, N-alkylated poly(acrylamides) have been extensively studied for their thermoresponsive behavior, such as PNIPAM, PNIPMAM, PNCPAL, PNEAM, PNAGA, PNCPAL and PNVCL [90]. The most popular poly(acrylamide) is PNIPAM, which has a 32–34 °C LCST with type II transition behavior [127,128,129]. Another important example of a water-soluble material with LCST properties is PMVE, which usually exhibits LCST values ranging between 33 and 37 °C with typical type III demixing behavior [130]. LCST properties were also found in polymers of natural origin, such as hydroxypropyl cellulose (HMC), methylcellulose (HPC) and elastine [130]. It should be noted that cloud points are affected by chain modification and end groups. Polyethylene glycol (PEG), also known as polyethylene oxide (PEO) at higher molecular weight (M_w_ > 20,000), is a highly biocompatible polyester used in biomedical applications with thermoresponsive properties. PEG methacrylate polymers, or PEGMA, possess a lower LCST that depends on the number of ethylene oxide units [90]. Poly(*N*-ethyl oxazoline)s represent another important class of polymers with a transition temperature that is too high for any biological applications, although their modification with other polymers has shown some potential for drug delivery [130]. Polymers such as PNIPAM have been extensively used in combination with pH-responsive polymers for the fabrication of double-responsive copolymers. PNIPAM-based copolymers usually exhibit LCST values close to physiological temperature. This ability has been demonstrated in combination with polymers that do not exhibit thermoresponsive properties close to the physiological range, such as polyvinylalcohol (PVA) or PEO, and even in combination with UCST polymers such as polyacrylamide (PAAM). Copolymerization or grafting is also an excellent strategy to bring the cloud point closer to human temperatures, such as for poly(acrylamide-co-acrylonitryle) (PAAM-co-PAN), poly(allylamine-co-allylurea) (PAA-co-PAU) or poly(vinylalcohol)-co-vinylbutyrate) (PVA-co-PVB). Another important class of thermoresponsive copolymers of biological industrial interest contains polaxamers, commercially known as Pluronics^®^, Synperonics^®^ or Kolliphor^®^, which consist of a three-block copolymer of PEO and polypropylene oxide (PPO). Polymers that exhibit both UCST and LCST have also been documented. Copolymers such as poly(p-dioxanone)-*g*-poly(vinylalcohol) (PPDO-*g*-PVA) have been studied for their ability to switch between UCST and LCST behavior. The ability to switch between UCST and LCST behavior is controlled by adjusting the hydrophobic/hydrophilic ratio by adjusting the degree of polymerization of one of the two building blocks [131].

Among UCST polymers, poly(acrylic acid) and poly(sulfobetaine) are two of the most studied examples for industrial and biological applications. UCST in water and water mixtures has been studied for drug delivery applications in linear and cross-linked polymers, as well as copolymers such as poly(acrylic acid) [125], poly(sulfobetaine)s and poly(oxazoline)s polymers [117,131,132]. A list of polymers that exhibit thermoresponsive behavior in water is provided in Table 3. The table does not correlate cloud points with the molecular length or concentration of the polymers. It is worth mentioning the that molecular weight, concentration and proportion between comonomers should be associated with every LCST and UCST value in order to ensure reproducibility. The description of the type of miscibility behavior (type I, II or III) provides another useful indicator, as it allows one to assess whether the cloud point will be influenced by the chain length, concentration or the presence of cosolutes. Furthermore, this information is crucial to control the positions of both LCST and UCST through synthesis.

Since it is impossible to prepare a perfectly monodispersed polymer, it is necessary to consider the intrinsic limit of the models, which allows us to predict the behavior of a polymer. During a LCST transition, polymers go through a fractional precipitation process in which various portions precipitate depending on their different structural characteristics. This is also valid for type II polymers, such as PNIPAM, whose behavior is substantially, but not entirely, independent of the molecular mass. Given the importance of experimental evidence, discrepancies can be found in the literature. This is the case for PMVE, whose LCST values are reported at 33 [133], 35 [134] and 37 °C [135]; or of the PEO, whose LCST values oscillate between 85 and 175 °C [130,133]. However, it is detrimental to consider that a difference of just one degree in the LCST of the polymer can make a huge difference from a biological point of view. Given the growing interest towards the use of thermoresponsive polymers in biomedical applications, the development of well-standardized procedures for LCST and UCST determination is required in order to compare different polymer preparations.

## 7. The Need for Standardization in LCST Measurements

### 7.1. The Importance of a Polymer’s Molecular Mass and Concentration

One of the most important aspects of the demonstration reported by Solc et al. for the description of a polydisperse polymer solution with a concentration-dependent interaction parameter *g*(*T*,*ϕ*) is the determining roles played by the molecular weight and polymer concentration in defining the critical miscibility behavior [110,111,112,113]. This seems to suggest the need to develop a standardized system for the creation of databases of the thermoresponsive properties of polymers. The list of thermoresponsive properties compiled by Wohlfarth in the Handbook of Chemistry and Physics provides an excellent example of how LCST and UCST should be reported. The list provides a very wide database of LCST and UCST values of thermoresponsive polymers that are related to different molecular mass and concentration ranges. Furthermore, the dataset includes information on the percentages of comonomers used for the synthesis of copolymers and their morphology [124].

The difficulty in defining standardized procedures for the definition of the LCST and UCST of polymers seems to be linked to the difficulty in creating a wealth of interdisciplinary knowledge. It is worth nothing that the sectorialization of scientific publications [143,144,145] and the “me to science” phenomenon [146] have resulted in an increasing number of publications on thermoresponsive polymers, while at the same time the explanation of the basic thermodynamic concept behind LCST and UCST behavior remains relegated to a few fundamental studies [96,98,104,110,111,112,113,147,148,149,150]. To date, the dependency of critical miscibility behavior on the molecular weight and polymer concentration tends to be underestimated in many studies evaluating the possible applications of thermoresponsive polymers in biological environments [151,152,153,154]. Conversely, it is essential to provide a detailed description of the polymer miscibility behavior in order to avoid cytotoxic effects that may arise due to the presence of the polymer in a globule state in physiological environment [155].

A frequently reported misconception relates to the LCST of PNIPAM and PNVCL, which is usually reported to be 32 °C. In fact, several specific conditions are required for both polymers to exhibit the same LCST, although PNIPAM and PNVCL exhibit opposite critical miscibility behavior. Accordingly, their LCST depends on very different conditions. PNIPAM belongs to type II and its main advantage is having a LCST that is almost completely independent of the chain length and other factors, such as the concentration, ionic force and the presence of cosolutes in the aqueous environment. Conversely, PNVCL has a very variable LCST (between 25 and 50 °C), as it belongs to type I, and its behavior in water can be described by the Flory–Huggins theory. Since these thermodynamic models have an intrinsically limited range of applications, an accurate description of the polymeric properties is required to ensure reproducibility. Moreover, it is essential to establish whether a critical behavior is “independent” or “substantially independent” of a certain factor. The tabulated values of LCST and UCST are determined in two-component systems composed of a polymer and solvent. However, when facing the complexity of biological matrices, one must expect substantial variation from the theoretical predictions. The knowledge of the thermodynamic behavior of a polymer is fundamental for any application in the biological field, and the models that are used to describe their behavior should be as complete as possible. According to these principles, temperature ranges provide a more accurate description of polymer behavior compared to single LCST or UCST values, as they account for small dependencies towards differences in molecular weight and concentration. The LCST of PNIPAM should be reported over a narrow temperature range (31–34 °C), which account for type II behavior. The small variation accounts for the fact that the length of the polymer has a minimal influence on the value of the LCST. In the case of “type I” polymers such as PNVCL, it is of crucial importance to consider that their solubility depends on the concentration and molecular mass to ensure the reproducibility of the synthesis process [112,156,157,158,159,160,161].

### 7.2. The Difference between LCST and Cloud Point

Another particularly important issue related to the standardization of LCST and UCST measurements is the methodology of their determination. Typically, the determination of the LCST relies on the definition of the transition temperature as the “cloud point”. However, some studies have pointed out the subtle difference between the term “cloud point” and LCST. The cloud point, or *T_CP_*, is referred to as any position of the binodal curve (Figure 3), including the LCST. At the *T_CP_*, the clouding of the solution is caused by the transition of the polymer to the collapsed aggregated state, which accounts for microscopic phase separation. Since the*T_CP_*can be located at any position in the binodal curve, its position is always dependent on the specific polymer concentration. Conversely, the LCST represents the minimum of the binodal curve, which is theoretically independent of the concentration at a specific polymer molecular weight. The term LCST is commonly mistaken for *T_CP_*, especially in biology studies [96,151]. However, it should be noted that the binodal curve does not coincide with the cloud point curve in the miscibility diagram. The difference between the binodal curve and the *T_CP_* curve is caused by the different kinetic aspects involved during polymer aggregation and phase separation [96]. Accordingly, the LCST point should be attributed to the point at which phase separation is observed, while the *T_CP_* point is associated with solution turbidity and the formation of heterogeneous milky phases [96,162]. The*T_CP_*of a polymer can be easily modified via copolymerization [163,164,165,166] and end group modification [160,161] to tune the hydrophilic–hydrophobic balance of the polymer chains. Moreover, polymer–polymer, polymer–solvent and solvent–solvent interactions can be changed by using different polymer concentrations [96,127,161,167] and ionic strengths [96,127,161,168] and by mixing different polymers [96,127,169], which eventually results in the modification of the *T_CP_*. While the fine control of *T_CP_* has resulted in the utilization of thermoresponsive polymers for specific applications, the difficult definition of the transition temperature has led to the inability to reproduce and compare the results between different research groups [96,161,170]. Moreover, the utilization of different techniques, such as UV-VIS, dynamic light scattering (DLS), turbidimetry, ^1^H-NMR spectroscopy and dynamic scanning calorimetry (DSC), can provide different values for the *T_CP_* of the same sample [96,142,161]. This section provides an overview of the main techniques used for LCST and *T_CP_* determination.

### 7.3. LCST and Cloud Point Determination

When the polymer undergoes a coil-to-globule or a globule-to-coil transition, the formation of aggregates results in an abrupt variation of the scattering properties of the solution, which usually results in visible turbidity. This allows for the determination of the cloud point *T_CP_* via scattering or spectroscopic techniques. UV-VIS spectroscopy and DLS represent two of the most common techniques for the determination of the *T_CP_* of a thermoresponsive polymer. However, these methods provide an “indirect” measurement of the *T_CP_*, as the value is extrapolated through the saturation of the instrument signal. Accordingly, *T_CP_* measurements that are performed via DLS or UV-VIS should be considered to be qualitative rather than quantitative.

**Figure 6:**
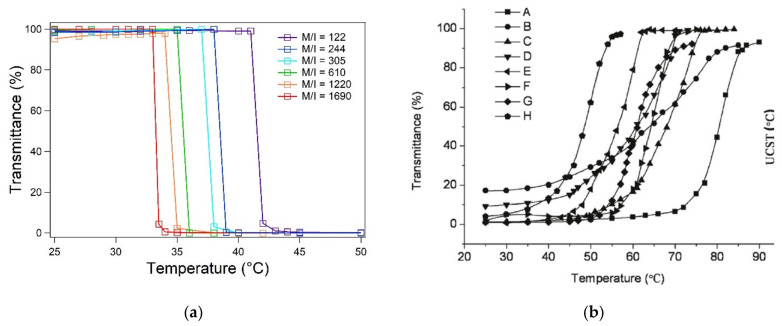
(**a**) Miscibility curves of PNVCL-COOH polymers (5.0 mg mL^−1^ aqueous solutions) with different molecular weights [161]. (**b**) Influence of structural parameters on temperature dependence of transmittance changes for 4.0 mg mL^−1^ aqueous solutions of PVA-PPDO copolymers. Reprinted with permission from [131]. Copyright 2011, American Chemical Society.

In UV-Vis measurements, the value of *T_CP_* is extrapolated from the curve of solubility, in which the transmittance of the solution is plotted against the temperature. The measurement should be carried out by choosing a wavelength (λ) in which the polymer does not exhibit an absorption band. Above the *T_CP_*, due to the cloudiness of the solution, the transmittance drops dramatically in a narrow temperature range. The presence of concentrated polymer aggregates scatters the incident light leading to a rapid decrease in solution transmittance. Upon cooling, phase remixing results in an increase in transmittance and the optical properties of the solution are restored. A common procedure is to associate the LCST of the polymer with the temperature in which transmittance drops to zero [151]. Conceptually, this is imprecise, as *T_CP_* represents an equilibrium temperature and should be associated with the inflection point of the miscibility (or solubility) curve (Figure 6a,b). The determination of LCST can be achieved by fitting miscibility curves with a simple sigmoidal model, as reported in Equation (17):

(17) $$y=Tr_{max}+\frac{\left(Tr_{max}-Tr_{min}\right)}{1+e^{\frac{\left(T-T_{CP}\right)}{dT}}}$$

in which *Tr* represents the transmittance and *T* is the temperature of the system. Thus, the two constants, *Tr_max_* and *Tr_min_*, are related to the optical properties of the systems before and after the transition [161]. The *T_CP_* related to LCST or UCST behavior corresponds to the value of the inflection point *T_CP_*. The model is suitable for the study of polymer behavior in complex matrices as well as in dilute regimens.

Turbidimetry represents one of the most common methods for the determination of the *T_CP_* of a thermoresponsive polymer. The measurement consists of measuring the loss of intensity of transmitted light due to the scattering effect of the polymer particles suspended in the solution. The output provides a miscibility curve (transmittance vs. temperature) in a similar way to *T_CP_* measurements performed with a UV-Vis spectrometer equipped with temperature control. However, the utilization of turbidimeters is a dynamic method, as the temperature can be constantly changed during the experiment. It has been reported that the temperature ramp can influence the results that are obtained throughout the experiment. To date, a heating rate of 1 K min^−1^ has become common to decrease the duration of the measurements. In cases of broad turbidity, the attribution of *T_CP_* to the inflection point of the curve may be subject to interpretation [96].

DLS measurements provide an estimation of the hydrodynamic diameter of particles in solution according to their scattering intensity. Accordingly, the LCST can be determined through a comparison of the particle size distribution at different temperatures. However, the characterization of polymer solutions via DLS requires specific molecular weight and concentration conditions, depending on the polymer under observation. Furthermore, data interpretation is complicated by the rapid aggregation of polymer globules that are formed during LCST transitions. Nonetheless, it is worth considering that the dimensional distributions of DLS are based on the use of mathematical models that may not be suitable for the description of the samples under analysis. A simpler approach is to consider the variations in scattering intensity as an analytical method for measuring the *T_CP_* via scattering techniques. This can be simply achieved by comparing the correlogram of polymer solutions at different temperatures. Since the correlogram from the sample above the *T_CP_* contains larger particles, the correlation of the signal takes longer to decay. According to Rayleigh’s approximation, the scattering intensity (I) has a strong dependency on the hydrodynamic diameter (D_h_) of the scattering object $I\propto D_{h}^{6}$. Compared to absorbance measurements, DLS is more sensitive upon the formation of polymer particles. UV-Vis allows the visualization of LCST transitions only when the concentrations of aggregates reach a point in which a displacement of the baseline is observed. In contrast, one single aggregated particle with a diameter of 1μm provides the equivalent scattering signal of 10^8^ polymer molecules with a diameter of 10 nm. Accordingly, the analysis of the same polymer solution using UV-VIS and DLS should provide different *T_CP_* values depending on the polymer polydispersity. Fractions with higher molecular masses will undergo the transition at lower temperatures and can be detected by DLS.

Since LCST is associated with the minimum of the binodal curve, the binodal of the phase diagram needs to be constructed in order to identify the minimum phase separation temperature. For aqueous solutions, this can be achieved by dissolving the polymer in water at low temperatures and annealing the solutions above the phase separation temperature. The point of the binodal curve can be constructed experimentally by changing the polymer concentrations and the temperature of solution.

**Figure 7:**
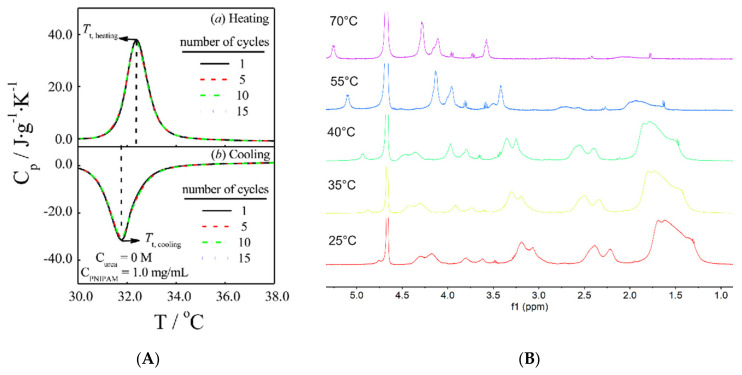
(**A**) Differential scanning calorimetry thermograms of PNIPAM aqueous solution (1.0 mg/mL) during four different heating−cooling cycles (cycles 1, 5, 10 and 15). Heating and cooling rates were both 1.00 °C/min. Reproduced with permission from Gao [171], copyright by ACS Publications, 2014. (**B**) ^1^H NMR spectra of chitosan-graft-poly-*N*-vinylcaprolactam in D_2_O recorded at different temperatures.

DSC is an analytical and dynamic method for the determination of LCST. DSC is a thermoanalytical technique in which the difference in the amount of heat required to increase the temperatures of a sample and reference is measured as a function of temperature. DSC provides the enthalpy of the phase transition, as well as the temperature at which the transitions occur. The coil-to-globule transitions of a LCST transition represent an endothermic process due to breakage of hydrogen bonds between the thermoresponsive polymer and the water molecules. Accordingly, DSC can provide an estimation of the number of hydrogen bonds involved in the LCST transition. If the number of the repeating unit is known, the number of water molecules leaving the polymer chains can be calculated from the energy of the transition peak. The thermograms of a PNIPAM aqueous solution are depicted in Figure 7A.

^1^H NMR is a spectroscopic technique that can provide insights into the thermoresponsive behavior of thermoresponsive polymers at the molecular level. When polymer-deuterated solutions are heated above their *T_CP_*, the decrease in chain mobility results in a partial dehydration of the polymer globules and in a decrease in the mobility of the polymer chains. Accordingly, the spectral line width increases and the peaks can disappear from the spectrum if the temperature is sufficiently above the *T_CP_*. If the polymer is highly hydrophilic, broad peaks will remain visible above the *T_CP_* due to the interactions with water molecules. Regarding copolymers, the two different moieties may exhibit opposite behavior, such as in the case of chitosan-graft-poly-*N*-vinylcaprolactam (Figure 7B). During the heating process, CS signals exhibit the expected behavior of a polysaccharide in solution. As far as the temperature increases, the absorption signals related to CS protons are deshielded and the band width is reduced due to reductions in viscosity and desolvation. On the contrary, PNVCL residues undergo LCST transitions and the signal intensity disappears and almost vanishes at 70 °C. While CS becomes more desolvated as the temperature increases, PNVCL becomes more hydrophobic. The desolvation of CS results in the reduction of the spectral linewidth of the proton signals. On the contrary, the coil-to-globule transition “hides” the protons of the PNVCL inside the internal parts of the globules.

## 8. Poly-*N*-Vinylcaprolactam

**Figure 8:**
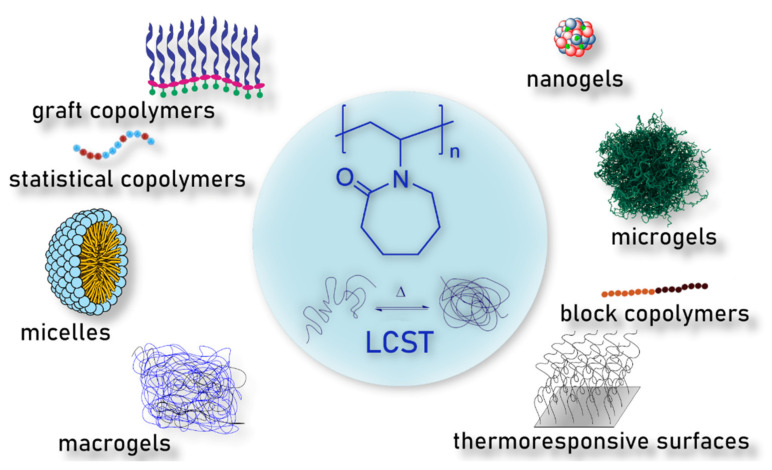
Schematic representation of PNVCL and its possible applications.

Poly(*N*-vinylcaprolactam) (PNVCL) is a temperature-responsive polymer that has been applied in biomedical applications, environmental applications, cosmetics and as an anticlogging agent in pipelines (Figure 8).

The polymer possesses exceptional film forming properties, is able to inhibit crystal growth and it interacts as a complexing molecule with organic substances. The monomer, NVCL, is an amphiphilic compound that is soluble in both polar and non-polar solvents. The first polymerization of NVCL was described by Shostakovski et al. and published in the Russian language in 1952 [172]. The synthesis and the bulk polymerization PNVCL was later described in English by Solomon et al. in 1968 [156]. The free-radical synthesis of PNVCL has been performed in many different solvents over the years, including benzene [173,174,175], toluene [156], isopropanol [149], DMF [151,153,154,176,177], DMSO and water mixture [178] and *p*-dioxane [159,179]. Recently, the synthesis of the polymer has been reported in water [158] and alcohol–water [180]. The polymer is soluble in both polar and non-polar solvents, such as alcohols, DMSO, DMF, THF, *p*-dioxane, chloroform, and dichloromethane. In water, the polymer exhibits thermoresponsivity and is soluble under its critical miscibility point (LCST). As previously mentioned, PNVCL is usually compared to PNIPAM for its thermoresponsive behavior, since both polymers exhibit LCST values close to the physiological temperature. However, their miscibility behaviors are completely different. PNIPAM belongs to type II and exhibits its LCST at ~32 °C, which is almost independent of the molecular weight, while PNVCL belongs to type I and its LCST depends on the molecular weight and polymer concentration [127,128]. The LCST of PNVCL is also lowered by the presence of salts [175,181,182,183]. It has also been reported that the addition of a small quantity of alcohol decreases the LCST in water [94,127]. Furthermore, the presence of compounds that increase the hydrophilicity of the polymer is known to increase the LCST [160]. In solution, PNVCL behaves as a polyelectrolyte and interacts with surfactants via hydrophobic interactions. Upon heating, the polymer undergoes a coil-to-globule transition upon reaching the critical point. During the transition, the shrinkage of the polymeric coils is accompanied by dehydration [127,128,157,184,185]. This has been observed through the reduction in the intrinsic viscosity near LCST [148]. The presence of anionic and cationic surfactants prevents the aggregation of the polymeric chains, thereby increasing the LCST [175]. This has also been reported for proteins, such as insulin and bovine serum albumin [186]. The type I critical behavior of PNVCL in water was confirmed and elucidated by Maeda et al. through infrared spectroscopy measurements [187]. The coil to globule transition in water was reported by light scattering [188], small-angle neutron scattering [189] (SANS) and fluorescence investigations [190]. The lack of popularity of PNVCL in relation to PNIPAAM has often been associated with the difficulty of polymerizing NVCL in a controlled fashion [94,127,128,184]. Since the thermoresponsivity of PNVCL is molecular-weight-dependent, the control of both the molecular weight and dispersity is of crucial importance [112,156,157,158,159,160]. In recent times, the possibility of controlling the polymerization and copolymerization of the monomer through controlled free-radical polymerization has resulted in an increasing number of publications. The polymer has also earned some fame due to its alleged biocompatibility, whereas many studies have addressed the fact that PNIPAM is inconvenient for biomedical applications [127,128,155,191,192]. The hydrolysis of PNVCL under acidic conditions has been described in detail by Imaz et al. [193]. Unlike PNIPAM, the cyclic amide group present in the structure of PNVCL prevents the formation of toxic amide compounds [155]. The utilization of strong acid provokes the opening of the lactam ring and produces a polymeric carboxylic acid and acetaldehyde as a side product. Despite the potential of the polymer for biomedical applications having been described for several years, the first “in vitro” evaluation of PNVCL cytotoxicity was published only in 2005 [155]. The study observed the behaviors of two cell lines, intestinal Caco-2 and pulmonary Calu-3, in relation to the exposure to PNVCL and PNVCL-grafted-PEO (PNVCL-*g*-PEO). The test demonstrated great cell tolerance towards the polymers, although at the same time it demonstrated that the polymer exhibits toxic effects above its LCST [155]. To date, the polymer has been tested in different cell lines, including human endothelial cells and different types of human carcinomas [127,194,195,196]. All studies have shown that the polymer is fundamentally biocompatible. Nevertheless, it is still necessary to assess the polymer’s toxicity under the conditions of specific biomedical applications. The biocompatibility of the polymer, combined with thermoresponsive properties, makes PNVCL a perfect candidate for biomedical and environmental applications. Due to its biocompatibility, the polymer has been commercialized as a hair setting product under the name Luviscol^®^ Plus [197]. Currently, the non-biodegradable nature of PNVCL is its major drawback. PNVCL absorbs numerous organic compounds from water [148,185,188,198]. As reported by Makhaeva et al., charged surfactants bind to PNVCL due to hydrophobic interactions [198]. In recent years, Rejinold et al. developed degradable nanoparticles with low toxicity and efficient cell uptake by combining PNVCL with CS [81,199].

Since PNVCL exhibits an LCST point in the proximity of physiological temperature, the polymer has been studied for applications in biochemistry and medicine. Due to its film-forming properties, the polymer has been studied for the fabrication of responsive surfaces. The polymer has been successfully applied for the bioseparation of proteins and tissue engineering [200,201,202,203]. The precipitation of trypsin using a thermoresponsive PNVCL-based membrane was first reported in 1992 [201]. Most of these applications rely on the ability to transform the polymer backbone into an insoluble state upon temperature exposure. Upon covalent modification, the polymer is covalently coupled with ligands that are specific to a target protein. By raising the temperature, the protein–polymer complex is precipitated. PNVCL was also investigated as a suitable environment for cell proliferation and manipulation. The implantation of PNVCL cryogels in C57B1/10 mice was studied by Shakya et al. [203]. The study demonstrated that the gel degraded rapidly and excluded local or systemic toxicities. Another aspect that makes PNVCL so interesting from a biomedical point of view is the possibility of forming thermoresponsive hydrogels. Hydrogels represent a class of materials that exhibit a three-dimensional and elastic network, which is formed from polymers crosslinked chemically or physically to form insoluble polymer matrices. In aqueous solutions, PNVCL hydrogels can swell or shrink as a response to a temperature change. PNVCL hydrogels have been successfully applied to cause the entrapment of various enzymes, such as trypsin and carboxypeptidase B, and for animal cell immobilization [127,128,204]. The hydrogel scaffold increases the stability of enzymes without affecting their enzymatic activity [204]. PNVCL-based hydrogels have been studied as systems for controlled drug delivery, especially in conjunction with other polymers. The utilization of copolymers containing PNVCL grafted onto polysaccharides has been reported for the preparation of hydrogels, microspheres, microgels and nanoparticles. The presence of a high content of functional groups in macromolecules such as CS, alginate or dextran has allowed the creation of many different biocompatible copolymers. The synthesis is usually started by generating free radicals on the polysaccharide backbone, which are subsequently used as macroinitiators for the monomers. In other cases, the two polymers are attached with a condensation reaction. In most of these syntheses, PNVCL is provided with a carboxyl group at the end of the polymeric chain [127,128]

## 9. Chitosan Thermoresponsive Copolymers

Polysaccharides such as CS represent an ideal matrix for the preparation of biocompatible copolymers that improve the biodegradability of synthetic thermoresponsive materials. In particular, CS is highly regarded for the fabrication of multi-responsive biopolymers due to its pH-responsivity and for the high reactivity of its functional groups. The groups involved in the reaction are the amino group in the C-2 position and the hydroxyls present in positions C-3 and C-6, respectively. It has been observed that the modification of CS is able to increase its solubility at neutral or basic pH [205,206]. The grafting procedure results in the formation of a graft copolymer characterized by a CS backbone and a series of branches (grafts) consisting of the synthetic polymer attached to the amino or hydroxyl group. Grafting is commonly achieved via a condensation reaction (*grafting onto*) between the end groups of the grafted polymer and the amino groups of CS (N-grafting) [207]. This procedure requires grafted polymers with a carboxyl terminal group, and the coupling reaction is generally accomplished using EDC and NHS as coupling agents. The *grafting onto* route has been praised for the high purity and defined structure of the resulting synthetic products. In the past years, “click chemistry”, as termed by Sharpless et al. [208,209,210], has revolutionized polymer chemistry due to its high specificity, quantitative yield, solvent insensitivity and mild conditions required [211]. On the contrary, O-grafting involves multi-step reactions and the utilization of less green reagents. The synthesis of O-grafted CS copolymers usually requires N-phtaloylation, followed by the reaction and regeneration of amino groups [212,213,214,215]. Nonetheless, such procedures allow one to further functionalize the polymer device. In recent times, multi-step procedures have been proposed for the fabrication of nanoparticle-conjugated thermoresponsive polymers [216] and for the N-conjugation of peptides with thermoresponsive copolymers for selective tumor cell recognition [215]. Another approach (*grafting from*) consists of the polymerization of the grafted polymer in presence of the CS backbone, which is chemically modified via the introduction of active sites. This synthetic approach has similarities to the synthesis of nanoparticles, as it considers CS macromolecules as objects in solution that are functionalized. According to their high molecular weight, which is usually between 10 and 1000 KDa, the behavior of a single CS molecule in solution may resemble that of the polymeric nanoparticles. Recently, studies have shown that some CS copolymers self-assemble in nanoparticles once dissolved in water [215]. Initially, the major drawback of *grafting onto* procedures was the lack of control of the polydispersity using FRP. In recent times, the utilization of atom transfer radical polymerization (ATRP) has provided an alternative for the synthesis of graft chains with controlled molecular weights [211,217].

### 9.1. Chitosan-graft-poly-N-isopropylacrylamide

**Figure 9:**
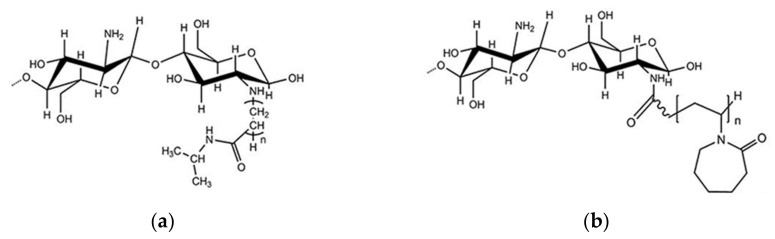
(**a**) Chemical structure of chitosan-*graft*-poly(*N*-isopropylamide). (**b**) Chemical structure of chitosan-*graft*-poly(*N*-vinylcaprolactam).

PNIPAM has been grafted onto CS with *grafting from* procedures using different initiators, including cerium ammonium nitrate [218], AIBN [219], potassium and ammonium persulfate [220,221,222,223]. The preparation of carboxyl-terminated PNIPAM has been documented for the preparation of thermoresponsive CS copolymers via the *grafting onto* approach [224,225,226]. The synthesis was first described by Chen et al. in 2006 and involved the utilization of 3-mercaptoacetic acid (MAA) as a chain transfer agent [226]. However, the lack of control of the chain length of PNIPAM has favored the utilization of ATRP. In 2010, Bao et al. reported on the synthesis of a CS-grafted PNIPAM (Figure 9a) with azide end groups [217]. An interesting combination of ATRP and click chemistry was later reported for the synthesis of a comb-like CS(-*g*-PDMAEMA)-*g*-PNIPAM terpolymer, which represented the first example of a dual-hydrophobic CS copolymer that switched from random coils to core–shell micelles, depending on pH and temperature conditions [211]. Cs-*g*-(PNIPAm) has been described as a potential pH- and thermoresponsive in situ gel-forming material for drug delivery [207]. CS-graft-PNIPAm hydrogels have been studied to increase the oral administration of drugs with low solubility, such as caffeine [227], naproxen [228] and paclitaxel [229], and to promote mucosal administration of hydrophobic drugs [230]. Nano- and microgels have been proposed for oncological applications for the delivery of curcumin [224] and 5-fluorouracil [223] and for antibacterial applications [231]. The utilization of fluorescent drugs enclosed in multifunctional inorganic materials consisting of Fe_3_O_4_ magnetic nanoparticles, CdTe quantum dots embedded in a mesoporous silica as the core and CS-*g*-PNIPAM as the shell has been proposed for theranostic applications [232,233]. Recently, the utilization of magnetic multi-responsive CS-graft-PNIPAM microgels has been proposed for vincristine delivery [234]. Rejinold et al. reported that CS-*g*-PNIPAM microgels exhibit specific toxicity towards PC3 cells [224]. The utilization of interpenetrated cryogel scaffolds of PNIPAM and CS has been proposed for bioartificial liver devices and for the purification of plasma [235].

### 9.2. Chitosan-graft-poly-N-vinylcaprolactam

The *grafting from* preparation of CS-*graft*-PNVCL (CS-*g*-PNVCL) copolymer (Figure 9b) was first described in a short communication by Kudyshkin et al. in 2007 [236]. The procedure had been previously used for the preparation of CS-*g*-poly-*N*-vinylpirrolidone [237] and CS-*g*-vinylacetate [238] and consisted of the radical polymerization of NVCL in the presence of CS using PPS as an initiator [236]. The procedure had many disadvantages, such as difficulties in controlling the length of the PNVCL and in the purification of the final product from the homopolymer [207]. In addition, it has been shown that the thermal decomposition of PPS promotes the cleavage of CS glycoside bonds [236,239]. The *grafting onto* approach for the synthesis of CS-*g*-PVNCL was reported by Prabaharan et al. in 2008 [151]. The procedure resembled the one reported by Chen et al. for the preparation of CS-*g*-PNIPAM [226] and was based on the utilization of AIBN as an initiator and 3-mercaptopropionic acid (MPA) as a termination agent. The procedure has been documented by many other authors [152,153,154,199,240,241,242] and even referred to as “pioneering” in a review by Argüelles-Monal et al. [207]. Despite this, the study of PNVCL’s properties and its characterization remains controversial and has contributed to passing down the misconception that PNVCL possesses a well-defined LCST at 32 °C [161]. In their introduction, Prabaharan describes PNVCL as a “*polymer with a lower critical solution temperature (LCST) at about 32 °C*”, while it is well known that PNVCL is a type I thermoresponsive polymer, with a LCST range that can vary greatly depending on the molecular mass and concentration of the PNVCL and the presence of ions and surfactants [127,128,161]. Furthermore, the reported molecular mass (1 kDa) appears to be too low in relation to the LCST value and no technical information is provided for the GPC-SEC analysis. In contrast to PNIPAM, mass characterization of PNVCL is crucial because of the relationship between the molecular mass and LCST. In addition, many authors have noted that the molecular mass determination of PNVCL using GPC-SEC is complicated due to sorption on the column in most solvents, including H_2_O [127,149,161] or THF [184,243]. The data are in strong contrast with the LCST–molecular mass dependence that was previously reported by Meeussen [111,112] and Kirsh [148]. According to Meeussen, PNVCL polymers should be at least 40 KDa in order to exhibit LCST at temperatures inferior to 35 °C [112]. A similar dependence has recently been demonstrated for PNVCL-COOH polymers prepared using the same procedure [161]. Accordingly, this misconception may have affected the reproducibility of subsequent works [170]. The first application in the field of nanotechnology of CS-*g*-PNVCL was reported by Rejinold in 2011 for the treatment of MCF7 and KB cell lines with 5-fluoruracil [81]. In the following period, Rejinold published few studies regarding the use of a series of PNVCL-based microgels [244], including CS-*g*-PNVCL formulations, for the treatment of breast cancer [199,224,240,241,242]. This led to a first in vivo study that demonstrated that CS-*g*-PNVCL can prolong the circulation of drugs such as curcumin and promote tumor localization [241]. Microgels were produced by ionotropic gelation, and the incorporation of drugs and inorganic nanoparticles was obtained by simply adding them into TPP solutions prior to gelation. In this way, Rejinold first described the fabrication of microgels loaded with gold [241,242] or magnetic Fe_3_O_4_ nanoparticles [240] for remote-controlled delivery of chemotherapeutic drugs. However, the recent retraction of a study originally published in 2014 raised some doubts about the legitimacy of the presented results [242]. The retraction was motivated by the duplication of six images, including results related to cytotoxicity tests, mice tumor growth and haemolysis tests [170,242]. One of the problematic aspects of Rejinold’s was particle characterization, and in particular size distribution. When it was presented, the interval of the size distribution provided by Reinold was between 100 and 300 nm, with the main peak of the histogram reaching only 1% of the total intensity [81,170,224,240]. Accordingly, the size interval represents only a small fraction of the analyzed samples. In 2017, Indulekha first described the remote-controllable elution of (DOX) from magnetic CS-*g*-PNVCL microgels using alternate magnetic fields [153]. This resulted in a step-like elution profile of DOX, which was also influenced by pH and temperature conditions. The study also reported that the gel dimensions increased above the LCST, suggesting a swelling behavior, as already hypothesized by Rejinold [81]. However, the choice to use DOX remains controversial, as it is known that the weak interaction between DOX and CS prevents the encapsulation of the drug [245,246]. Nonetheless, it should be pointed out that drug encapsulation may be promoted by the aggregation processes that takes place during freeze-drying [247]. Rampino noted that the lyophilization leads to an increase in the size of CS-based nanoparticles, even in the presence of cryoprotectans such as sucrose or trehalose [247]. The study by Rampino suggested the need to redefine the standards for elution tests, as freeze-dried particles exhibit different size and physicochemical properties that can eventually affect the elution profile of the encapsulated drug. Indulekha also reported the fabrication of another Au NPs/CS-*g*-PNVCL hybrid device, which consisted of Au NPS nucleated directly on the surface of CS-*g*-PNVCL [152]. The study demonstrated the reduction of the toxicity of Au NPs in both L929 and MC-F cells and suggested the utilization of these devices as theranostic nanoprobes for image-guided triple therapy consisting of photothermal, chemo and radiotherapy treatments. A different approach was described by Banishem et al, who reported the fabrication of an Au NP/CS-*g*-PNVCL hybrid using thioglycolic acid as a ligand for the stabilization of Au NPs during ionotropic gelation [248].

In recent times, the synthesis of new multi-functional CS-*g*-polymers has been described. Durkut reported on the synthesis of a CS-*g*-galactosilate-*g*-PNVCL triblock polymer that exhibited pH- and thermoresponsivity [249]. A brilliant strategy for the preparation of CS-*g*-PNVCL conjugated with a peptide for selective recognition of breast cancer was described by Niu et al. [215]. The study reported a multi-step synthesis approach involving the protection of amine residues through the phtaloylation [212] and conjugation of PNVCL via reversible addition fragmentation (RAFT) using *S*-1-dodecyl-*S*′-(α,α′-dimethyl-α″-acetic acid) trithiocarbonate (DDACT). The resulting polymer loaded with DOX self-assembled into 200 nm microparticles in water. The results *in-vivo* and *in-vitro* on MCF-7 cells and xenographted mice demonstrated that the device increased tumor accumulation with a substantial reduction in DOX toxicity, which resulted in prolongation of the life span of the treated animals [215]. Another *grafting from* approach was reported by Sahebi, which consisted of the polymerization of NVCL in the presence of Fe_3_O_4_ nanoparticles, which were subsequently conjugated with CS using EDC/NHS [216].

CS-*g*-PNVCL-related systems are starting to show some promising properties in environmental applications. In 2019, Bahmani developed CP/ZIF-8 (zeolitic imidazolate framework) nanofibers for the removal of As (V) and Cr(VI) from water. More recently, a tri-block polymer comprising PAA, PNVCL and CS was developed and used as a biocompatible flocculant for water remediation [250]. The polymer was efficiently applied for the removal of turbidity, ciprofloxacin and Cd(II) from water. The study showed that the ability to bind pollutants in the polymer increased above the LCST.

### 9.3. Other Thermoresponsive Chitosan Polymers

Click chemistry allows the combination of living or controlled radical polymerization with organic coupling procedures. Accordingly, this has enabled polymer scientists to combine CS with one or multiple polymers via grafting procedures. The possibility of combining multiple polymers has allowed conditions to be reached in which the physical and chemical properties of one polymer balances out the limitations of another polymer present in the polymer blend.

Over the years, different thermoresponsive formulations have been proposed using CS in combination with oligo(ethylene glycol) methacrylate (OEGMA). The synthesis of a comb-shaped copolymer of chitosan-*g*-OEGMA was approached using both *grafting from* and *grafting onto* approaches using ATRP by Munro et al. [251]. Thermoresponsive CS-graft-P(2-(2-methoxyethoxy)ethyl methacrylate-co-OEGMA) copolymer (CS-*g*-p(MEO_2_MA-co-OEGMA)) was synthesized via a *grafting onto* approach by Li et al. [252]. The procedure included the protection of CS amino groups via the formation of an alkynyl CS derivative. The polymerization of MEO_2_MA and OEGMA was carried out via ATRP and the MEO_2_MA-co-OEGMA copolymer was grafted onto CS via click polymerization using CuBr/*N*,*N*,*N*′,*N*″,*N*″-pentamethyldiethylenetriamine (PDMETA) as the catalyst. The resulting polymer self-assembled into micelles and exhibited tunable and reversible LCST behavior in water. The LCST was controlled by modifying the molar ratio of MEO_2_MA and OEGMA [252]. The heterogeneous grafting of CS with thermoresponsive OEGMA)/MEO2MA)/acrylonitrile (AN) was reported by Kwan et al. The synthesis was accomplished using the *grafting from* approach. CS was modified with acryloyl chloride and the resulting polymer reacted with SG-2 based BlocBuilder, which was used as the initiator. The thermoresponsive terpolymer was grafted in the presence of the modified CS using nitroxide-mediated polymerization (NMP) [253]. Den et al. reported on the preparation of magnetothermoresponsive micelles using a star block copolymer of poly(ε-caprolactone)(PCL)-block-MEO_2_MA-co-OEGMA and Mn- and Zn-doped ferrite magnetic nanoparticles for the delivery of DOX. The star block copolymers exhibited LCST at 43 °C and exhibited high biocompatibility in HepG2 cells [254]. Naik et al. reported the utilization of CS and glycerophosphate thermoresponsive gels for the administration of doxepin. The administered gels showed a gradual increase in activity due to the increase time residence of the drug in the nasal cavity. A recent study by Shou et al. reported the design and preparation of catechol-hydroxybutyl CS (HCBC-CS) via the *grafting onto* approach. The polymer was used for the fabrication of injectable multifuctional hydrogels that exhibited VPTT at different temperatures and were used as hemostatic agents [255].

## 10. Conclusions and Future Perspectives

A wide number of natural and synthetic polymers have been proposed for use in the production of drug delivery devices for biomedical applications. Polymers of natural origin, such as CS, are highly regarded for their biocompatibility and biodegradability, although their utilization is limited by their batch-to-batch variability. On the other hand, the properties of synthetic polymers are easily controlled via the polymerization settings. While this tunability has led to an increase in their utilization in different fields, their biological application is still hindered by their low biodegradability. Thermoresponsive synthetic polymers such as PNVCL or PNIPAM have been extensively applied in biomedical and environmental applications due to the possibility of tuning their thermoresponsive properties. The polymers experience a coil-to-globule transition above a critical temperature (LCST) due to the abrupt change in their hydrophobic–hydrophilic equilibrium. The combination of PNVCL or PNIPAM with CS via the grafting approach has increased the biodegradability of the thermoresponsive polymers. CS thermoresponsive polymers have been applied for the preparation of sensors and in oncological and environmental applications. In recent times, the combination of thermoresponsive CS with inorganic nanoparticles has allowed the possibility of controlling the drug release remotely. Moreover, the modulation of the transition temperature remains difficult in in vivo models, which is still a limitation in the rapid translation of these systems to the clinic. Despite the increasing number of researchers that are working on LCST polymers, the fundamentals of the LCST phase transition behavior still needs to be clearly acknowledged. For this reason, it is still difficult to compare the thermoresponsive behaviors and LCST and *T_CP_* values of polymers across different studies. It is fundamental to standardize data reporting in order to provide more detailed information regarding the analyzed polymers, by providing polymer concentration and molecular weight information. Furthermore, it has been shown that the determination of the transition temperatures results in differences depending on the methods used.

## Figures and Tables

**Table 1:** List of polymeric biomaterials related to their sources and applications.

Source	Name	Structure	Biological Activity
Plants	Cellulose	β-(1→4) linked D-glucopyranose	Bowel movement regulator, stool bulk increaser
	Hemicellulose	Xylans, mannans, mixed linkage β-glucans and xyloglucans	Bowel movement and cholesterol level regulator, free radicals scavenger, immunomodulator and antithrombotic
	Starch	α-(1→4) and/or (1→6) linked d-glucopyranose	Prebiotic agent, regulation of blood glucose levels, enhancement of mineral absorption, prevention of colorectal cancer
	β-glucans	β-(1→4) and β-(1→3) linked d-Glucopyranose	Cholesterol and blood glucose levels regulator, immunostimulator, antihypertensive
	β-glucans	β-(1→4) and β-(1→3) linked d-Glucopyranose	Cholesterol and blood glucose levels regulator, immunostimulator, antihypertensive
	Glucomannan	β-(1→4) linked-d-glucopyranose and β-(1→4)-linked d-mannose	Cholesterol level regulator, anticonstipation agent
	Inulin	β-(1→2) linked d-Fructofuranose	Hypolipidemic and prebiotic agent, mineral absorption enhancer
	Pectins	α-(1→4) linked d-galacturonic acid and rhamnose backbone, arabinose, galactose, xylose side chains, partially *O*-methyl/acetylated	Cholesterol level regulator, intestinal immunomodulator, gastric and small-intestine and cholesterol-lowering effects, gastric emptying decreaser
	Guar, Arabic and locust bean gum	Galactan, xylan, xyloglucan, glucuronic mannan, galacturonic rhamnosan type	Hypocholesterolemic and hypotriglyceridemic agent, gastric emptying decreaser
Animals	Chitin and chitosan	β–(1→4) linked d-glucosamine, partially *N*-acetylated	Tablet component, absorption-enhancing agent
	Hyaluronic acid (hyaluronan)	β-(1→4) and β-(1→3) linked glucuronic acid and *N*-acetylglucosamine	Useful in cancer, wound repair, inflammation, granulation, cell migration, skin healing, fetal wound healing
	Heparin	2-*O*-sulphated-α-l-iduronic acid, β-d-glucuronic acid and *N*-sulfated or 6-*O*-sulfated-α-d-glucosamine	Anticoagulant, used in cancer treatment, tissue engineering and biosensors
Algae	Alginates	Β-(1→4) linked d-mannuronate and α-l-guluronate	Controlled drug release, cells encapsulation, tissue engineering and for preparation of dental molds
	Carrageenan	α-(1→4) and β-(1→3) linked d-galactose and d-anhydrogalactose, partially substituted by ester sulphates	Buccal, ophthalmic and vaginal drug delivery systems
	Red algae sulphated polysaccharides, porphyran,	Backbone of alternating β-(1→3) linked d-galactosyl units and α-(1→4) linked l-galactosyl, (1→6) 3,6-anhydro or sulphate-α-l galactosyl units	Antiviral (herpes simplex virus types 1 and 2)
	Green algae sulphated polysaccharides	(1→3) linked galactose, (1→3) linked arabinose, partially 6-*O* and 3-*O* sulphated, (1→4)-linked glucopyranose and terminal (1→4)-linked xylose	Antioxidant and anticoagulant
Microorganisms	Dextran	α-(1→6) linked d-Glucopyranose with α-1,3 branches	Plasma expander
	Pullulan	α-(1→4) and α-(1→6) linked glucan or maltotriose	Anticoagulant and plasma expander
	Xantan gum	α-(1→3) linked glucopyranose backbone with trisaccharide side chains containing d-mannose-β-(1→4)-d-glucuronic acid-β -(1→2)-d-mannose	Carrier for drug and proteins
	Gellan gum	d-glucopyranose-β-(1→4)-d-glucoronic acid- β-(1→4)-glucopyranose- β-(1→2)-l-rhamnose α-(1→4)	Multifunctional excipient for pharmaceutical formulation

**Table 2:** Molecular weight dependency of LCST based on the type of miscibility behavior.

Miscibility Behavior	LCST Dependence on Molecular Weight
Type I	Dependent
Type II	Independent
Type III	Dependent for diluted solution, independent for concentrated solution

**Table 3:** List of polymers with thermoresponsive properties.

Name	Structure	UCST (°C)	LCST (°C)	Ref.
Hydroxypropyl cellulose (HPC)	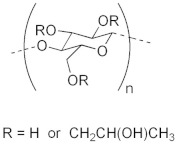		45.3–58.1	[124]
methylcellulose	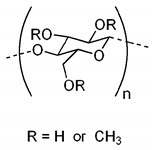		51.6	[124]
poly(acrylamide-co-acrylonitrlyle) (PAAm-co-PAN)	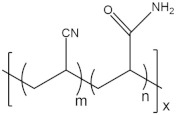	6.4–57		[136]
poly(allylamine-co-allylurea) (PAA-co-PAU)	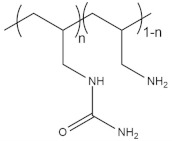	25–54		[100,137]
poly(*p*-dioxanone)-*g*-poly(vinylalcohol) (PPDO-*g*-PVA)	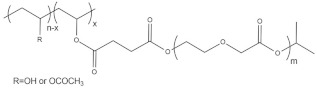	30–80	30–80	[131]
Poly(ethylene oxide) (PEO)	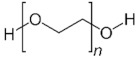		100–175	[133]
Poly(ethylene glycol) methacrylate (PEGMA/OEGMA)	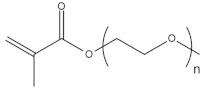		26–90	[138]
poly(ethylene oxide)-b-poly(propylene oxide)-b- poly(ethylene oxide (PEO-PPO-PEO or Pluronics or Polaxamer)	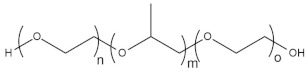		12.5–52.5	[135]
poly(hydroxyethylmethacrylate) (PHEMA)	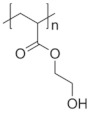	22		[133]
poly(methacryl amide) (PMAAm)	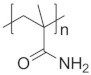	60		[133]
Poly(propylene glycol)	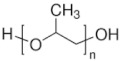		15–42	[133]
poly(vinyl methyl ether) (PMVE)	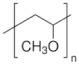		33–37	[124,133,135]
poly(vinylalcohol) (PVA)	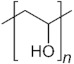		241	[124]
Poly(vinylalcohol)-co-vinylbutyrate) (PVA-co-PVB)	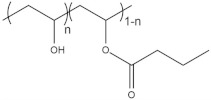	131	25	[124]
poly[2-(dimethylamino)ethyl methacrylate] (PDMAEMA)	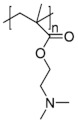		32–53	[139]
poly-2-isopropyl-2-oxazoline (PiPrOx)	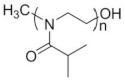		26–34	[140,141]
Poly-3-dimethyl(methacryloyloxyethyl)ammonium propane sulfonate (PDMAPS)	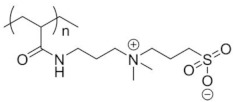	60		[133]
(PNAGA)	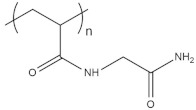		22	[133]
Poly(6-(acryloyloxymethyl) uracil) (PAU)	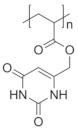	80		[133]
Poly-*N*-cyclopropylacrylamide (PNCPAL)	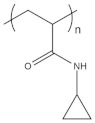		40–50	[135]
Poly-*N*-ethylacrylamide (PNEMAM)	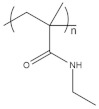		82	[133]
poly-*N*-isopropylamide (PNIPAM)	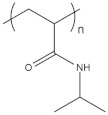		32–34	[124,127,142]
poly-*N*-isopropylmethacrylamide (PNIPMAM)	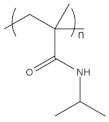		42–46	[135]
Poly-*N,N*-diethylacrylamide (PDEAAm)	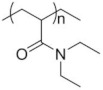		25–32	[90]
Poly-*N*-vinylcaprolactam (PNVCL)	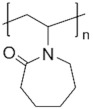		25–50	[112,127,128]

## Data Availability

The data presented in this study are available on request from the corresponding author.
